# Epidural Tramadol, is it a Good Option for Cesarean Section?

**DOI:** 10.5812/kowsar.22287523.2105

**Published:** 2011-09-26

**Authors:** Luciana de Souza Cota Carvalho

**Affiliations:** 1 Unifenas University of Medical Sciences, Minas Gerais, Belo Horizonte, Brazil

**Keywords:** Epidural anesthesia, Tramadol, Cesarean section

*Dear Editor*,

I read the article entitled “The Maternal and Neonatal Effects of Adding Tramadol to 2% Lidocaine in Epidural Anesthesia for Cesarean Section” (1). The authors demonstrated that tramadol 50 mg or 100 mg associated to lidocaine is appropriate for this surgery and also improve sensory and motor blockade effects without any increase in complications. However some important reflections and some missing data may be consider for full understanding of the article. For instance, one question came to my mind during the analysis-why haven’t the article focused on tramadol applied on spinal anesthesia? Neuraxial anesthesia is undoubtedly the most appropriate technique for elective cesarean delivery since the relative risk of mortality from general versus regional anesthesia is 16. 7 (2). General anesthesia in obstetric patients is a procedure with higher rates of serious and life-threatening complications, mainly related to airway management once the physiologic changes of pregnancy determine higher incidence of pulmonary aspiration of gastric contents and difficult airway (3,4). Until 1980, epidural anesthesia was the primary choice of neuraxial technique for cesarean delivery because spinal anesthesia was associated with a high risk of postdural puncture headache. After this period, the introduction of pencil-point spinal needles and several other advantages, the single-shot spinal anesthesia became the technique of choice for cesarean (5, 6). Besides that, tramadol intrathecal have been used with success for labor and major gynecological surgeries (7, 8).

Other important comments when reflecting about the results section, I was wondering the following points could have been provided to allow fully result analysis:

1. The mean dose of lidocaine and sufentanil request intraoperative were presented, but it was not showed the number of patients that demanded complementation analgesia and when;
2. Around 30% of patients present some complications but they did not mention what kind of complications and these numbers is relatively bigger than other found on literature (9) and
3. They also comment that general anesthesia is an option when maternal apnea lasts longer than 20 seconds or enable to speech or if the mother lost consciousness or did not respond to stimuli but they did not comment if some patients need it, considering the risks of general anesthesia, it is an important date.

Finally, the article core idea is very interesting, since epidural tramadol has comparable analgesic efficacy with lower side effects than epidural morphine, specifically concerning about respiratory depression (10). Although, this article still demands further improvements.
